# Immunoglobulin G4-associated cholangitis mimicking cholangiocarcinoma treated by laparoscopic choledochectomy with intracorporeal Roux-en-Y hepaticojejunostomy

**DOI:** 10.1186/1477-7819-12-363

**Published:** 2014-11-29

**Authors:** JiaQin Cai, Yi-Ping Mou, Yu Pan, Ke Chen, Xiao-Wu Xu, YuCheng Zhou

**Affiliations:** Department of General Surgery, Sir Run Run Shaw Hospital, School of Medicine, Zhejiang University, 3 East Qingchun Road, Hangzhou, 310016 Zhejiang Province China

**Keywords:** IgG4-associated cholangitis, Hepaticojejunostomy, Intracorporeal, Laparoscopic

## Abstract

Immunoglobulin G4 (IgG4)-associated disease is a recently recognized disease entity that is characterized by elevated serum IgG4 concentrations, abundant IgG4 lymphoplasmacytic infiltration, and dramatic steroid responses. IgG4-associated cholangitis is one manifestation of IgG4-associated disease. However, it is clinically challenging to make a preoperative differentiation between this rare disease and cholangiocarcinoma, especially for those with serum concentrations of IgG4 in the normal range. This article reports on a 57-year-old man with jaundice and upper abdominal discomfort. Imaging examination showed biliary stricture that closely resembled cholangiocarcinoma, and the patient’s serum IgG4 concentration was normal. The patient underwent a laparoscopic choledochectomy with Roux-en-Y hepaticojejunostomy using an intracorporeal hand-sewn technique. He recovered quickly without any complications. We also present our experience in laparoscopic intracorporeal hand-sewn hepaticojejunostomy.

## Background

IgG4-associated systemic disease (ISD) is a systemic disorder involving multiple organs associated with increased IgG4 serum levels or IgG4 positive plasma cell infiltrates. IgG4-associated cholangitis (IAC) is one manifestation of ISD. However, the clinical, biochemical, and imaging features of IAC mimic cholangiocarcinoma, making preoperative accurate diagnosis difficult [1]; therefore, surgery remains a therapeutic choice, so as to avoid omitting malignancy.

Since the development of minimally invasive surgical approaches [2], laparoscopic surgery for biliary tract diseases has evolved rapidly over the past decade, and laparoscopic Roux-en-Y cholangiojejunostomy and hepaticojejunostomy are accepted for the treatment of such diseases. On the basis of our extensive laparoscopic experience gained from laparoscopic gastrectomy and pancreatectomy, as well as other laparoscopic operations [3–6], we were encouraged to develop an intracorporeal hand-sewn technique for reconstruction. Herein, we report a case of IAC preoperatively diagnosed as cholangiocarcinoma and successfully treated by laparoscopic hepaticojejunostomy with our intracorporeal hand-sewn technique.

## Case presentation

A 57-year-old man was admitted to our department; he had jaundice and had been experiencing upper abdominal discomfort for the previous month. Laboratory data were as follows: alkaline phosphatase, 303 U/l; alanine aminotransferase, 266 IU/l; aspartate aminotransferase, 70 IU/l; γ-glutamyltransferase, 555 U/l; total bilirubin, 126 μmol/dl; direct bilirubin, 102.0 μmol/l. The patient’s serum levels of carcinoembryonic antigen and α-fetoprotein were within normal limits; however, serum carbohydrate antigen 19-9 concentration was 306 IU/ml. Other test results, including IgG4 serum levels, were all within normal ranges.

**Figure 1:**
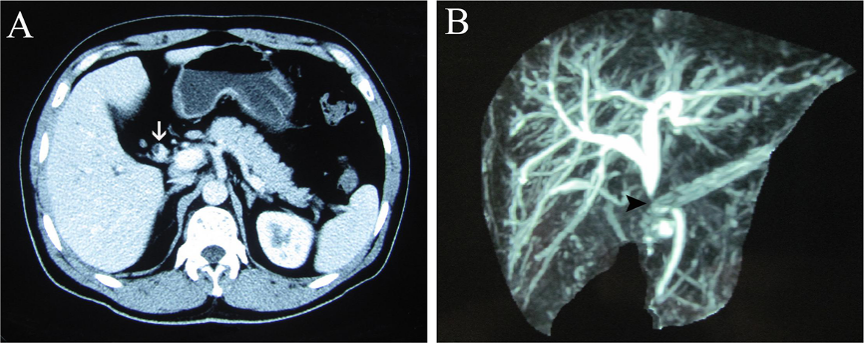
**Preoperative imaging examination. (A)** Computed tomography showed biliary stricture (white arrow). **(B)** Magnetic resonance cholangiopancreatography revealed biliary stricture (black arrow) and dilatation at the top of the stricture.

Enhanced abdominal computed tomography revealed a mass involving the common hepatic duct (Figure 1A). Magnetic resonance cholangiopancreatography revealed a stricture at the upper middle segment of the common hepatic duct, together with proximal bile duct dilatation (Figure 1B). The mass was initially diagnosed as a cholangiocarcinoma, resulting in biliary stenosis and jaundice. Therefore, laparoscopic choledochectomy with Roux-Y hepaticojejunostomy was selected.

**Figure 2:**
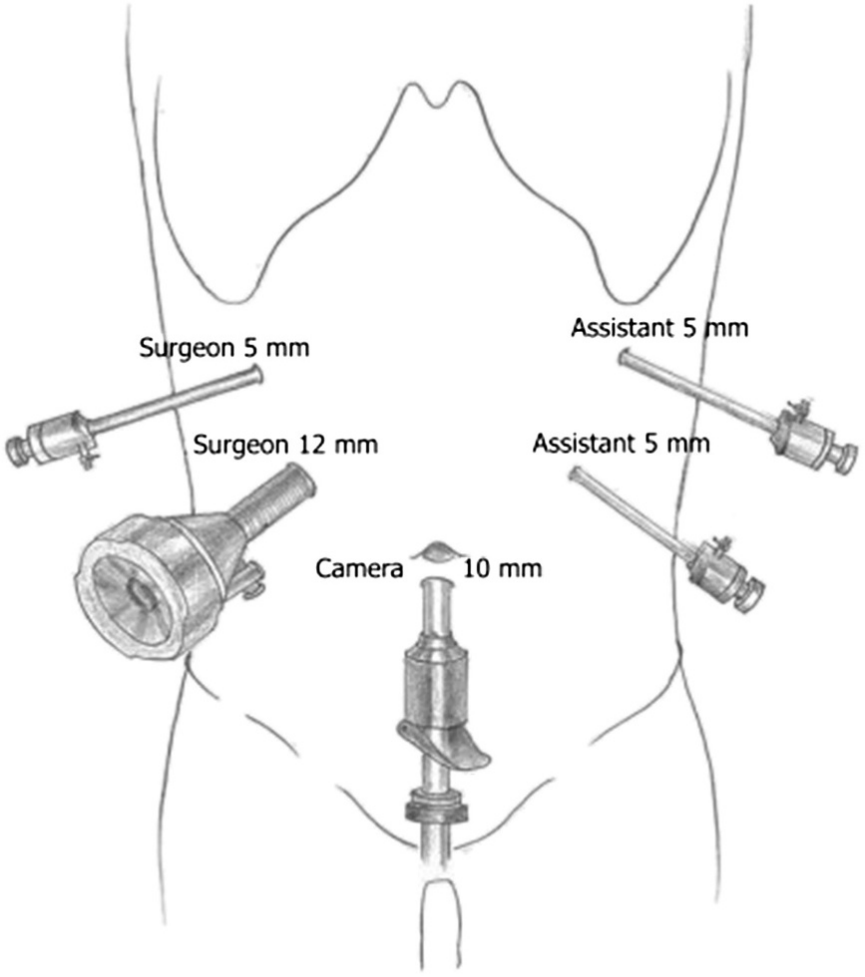
**Trocar placement.**

**Figure 3:**
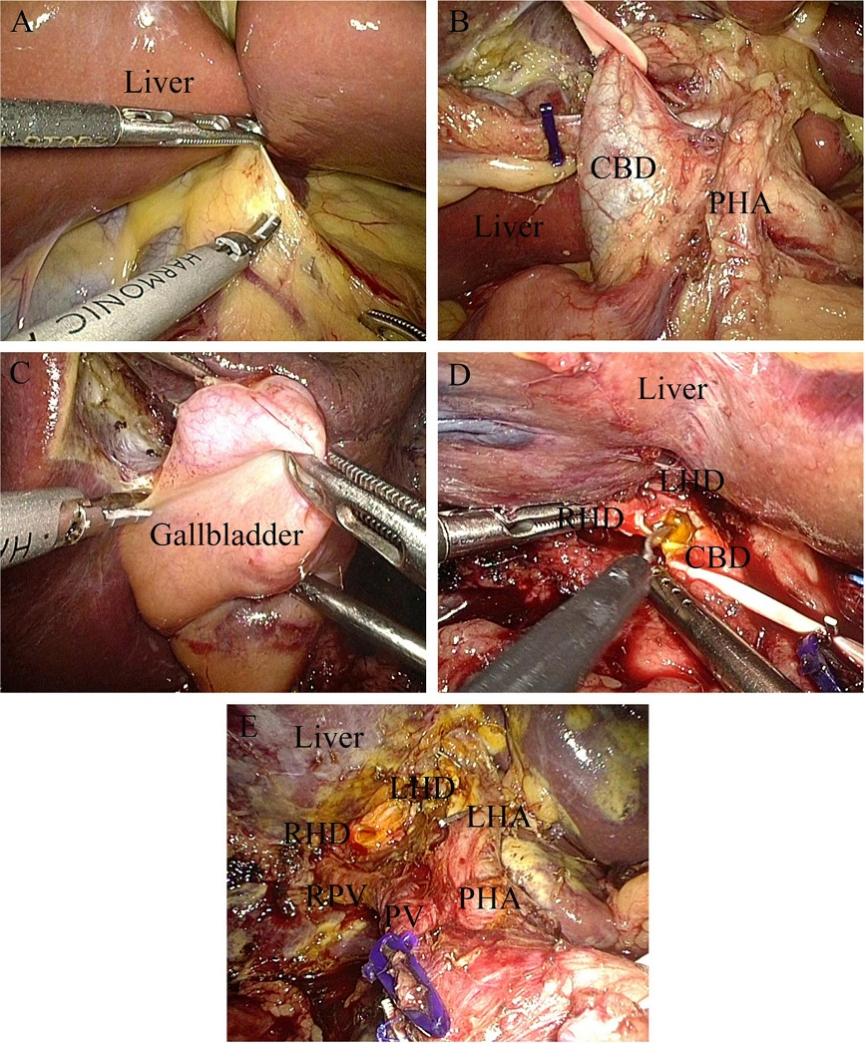
**Resection of mass on biliary duct. (A)** Division of hepatoduodenal ligament. **(B)** Visualization of properhepatic artery, common bile duct, and portal vein. **(C)** The cystic artery was clipped and divided while the cystic duct was clipped and left in situ. **(D)** Transection of left and right hepatic ducts 1.0 cm above the mass. (**E)** Traversal of common bile duct about 0.5 cm below the mass. CBD, common bile duct; LHA, left hepatic artery; LHD, left hepatic duct; PHA, proper hepatic artery; PV, portal vein; RHD, right hepatic duct; RPV, right portal vein.

The patients’ position and the placement of trocars were similar to our previous studies [7] (Figure 2). The hepatoduodenal ligament was divided by ultrasonic coagulating shears (Figure 3A). Then the common hepatic artery, common bile duct and portal vein were visualized (Figure 3B). Calot’s triangle was identified and the cystic artery was clipped and divided while the cystic duct was clipped and left in situ (Figure 3C). The biliary tract, including the common bile duct, common hepatic duct and left and right hepatic ducts was divided further from the portal vein. The portal vein and common hepatic artery were then separated from the surrounding tissue upward to the hilar plate. Lymphadenectomy around the pancreatic head was performed. The mass was found at the middle of the common bile duct. The dilated left and right hepatic ducts were transected 1.0 cm above the mass (Figure 3D), and the common bile duct was traversed about 0.5 cm below the mass (Figure 3E). The resected tissue and the gallbladder were taken out in an endoscopic retrieval bag through the umbilical incision.

**Figure 4:**
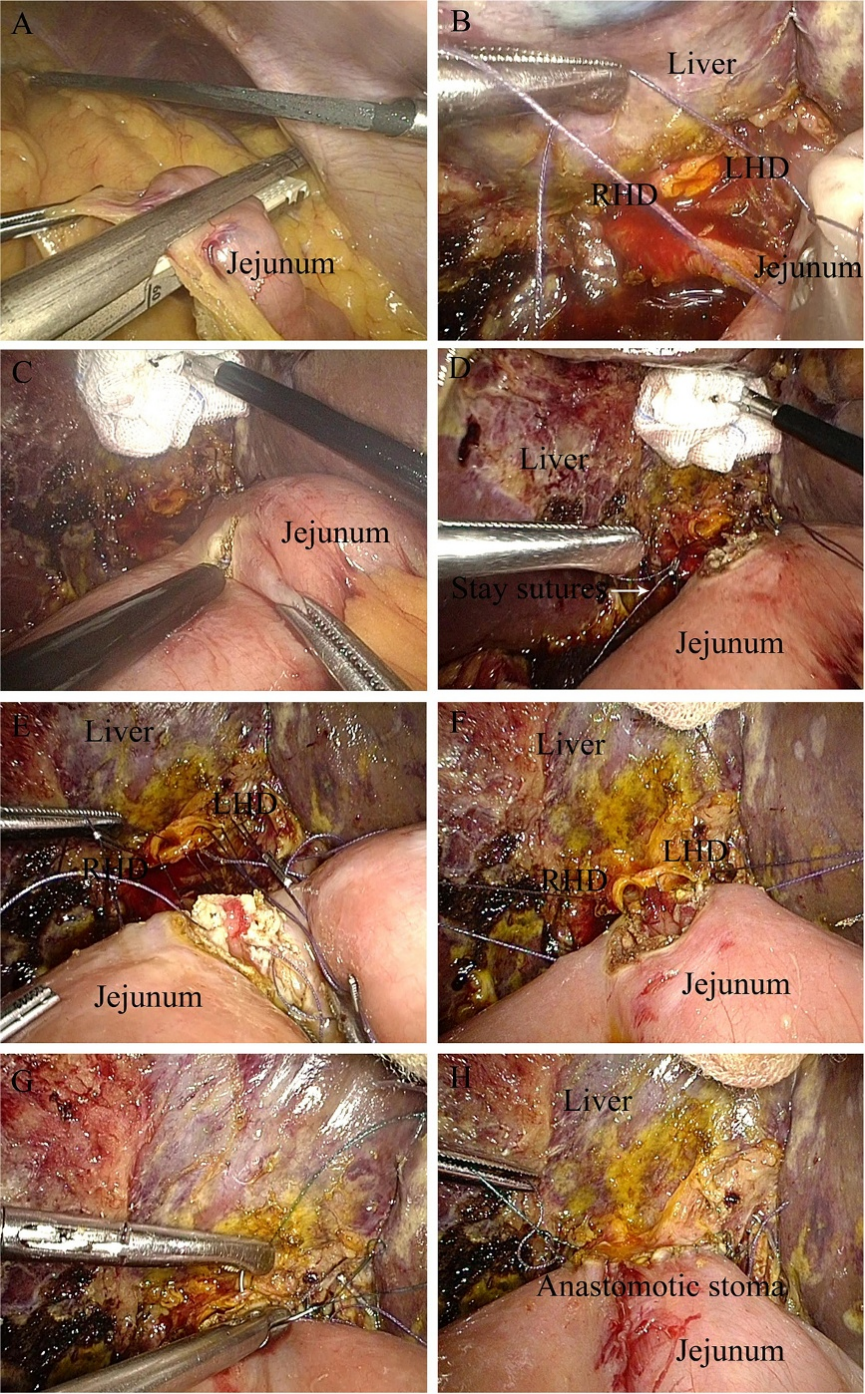
**Intracorporeal side**-**to**-**side jejunojejunostomy. (A)** Transection of the jejunum with an endoscopic linear stapler. **(B)** Approximation of left and right hepatic ducts and distal limb using a draft line. **(C)** A 5 mm wide incision at the antimesenteric side of the jejunum. **(D)** Long stay sutures to lift the corners at the 3 o’clock and 9 o’clock positions of the anastomotic stoma. **(E,F)** Suture of the posterior wall using interrupted sutures **(G)** Suture of the anterior wall using a continuous suture. **(H)** Strengthening of the seromuscular layer with interrupted sutures to reduce tension. LHD, left hepatic duct; RHD, right hepatic duct.

The incision was sutured, and the pneumoperitoneum was reestablished. After exploring the upper jejunum about 25 cm distally from the ligament of Treitz, an incision was made in the mesentery of this loop, and a transection of the jejunum was performed with an endoscopic linear stapler (Figure 4A). The left and right hepatic ducts and the distal limb were then approximated using a draft line (Figure 4B), and a 5 mm wide incision was made at the antimesenteric side of the jejunum (Figure 4C) for end-to-side hepaticojejunostomy. At the 3 o’clock and 9 o’clock positions of the anastomotic stoma, the sutures were held as long stay sutures to lift the corners (Figure 4D). The posterior wall of the hepaticojejunostomy was sutured using interrupted sutures (Figure 4E,F) and the anterior wall was sutured using a continuous suture (Figure 4G). The seromuscular layer was strengthened with interrupted sutures to reduce tension (Figure 4H). Then a side-to-side jejunojejunostomy was performed through the enlarged umbilical incision. Finally, a peritoneal cavity drainage tube was placed posterior to the bilioenteric anastomosis.

**Figure 5:**
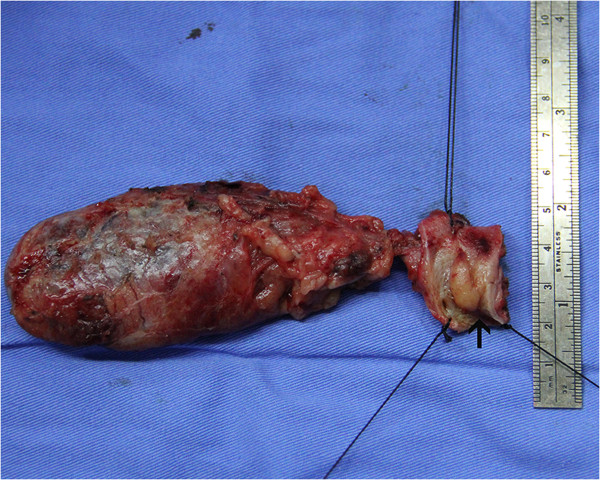
**Surgical specimen, showing a mass in the common bile duct.**

**Figure 6:**
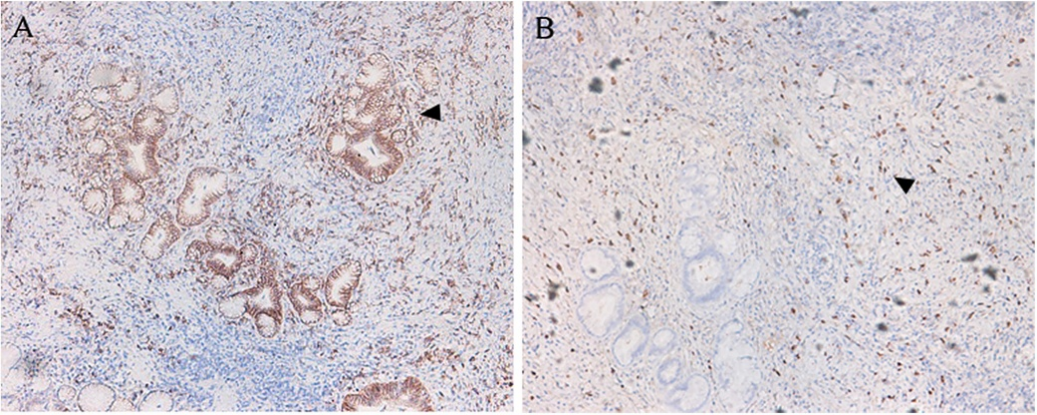
**Immunostained bile duct specimens. (A)** Moderate CD138-positive plasma cell infiltration, ×100. **(B)** Abundant IgG4-positive plasma cells, ×100.

The operative time was 210 min and blood loss was 60 ml. The intraoperative frozen pathological diagnosis was probably IAC, rather than cholangiocarcinoma, and the margins were negative. The gross finding was a 20 × 15 mm mass located in the middle part of the common bile duct, in communication with a dilated proximal bile duct (Figure 5). Microscopically, lymphoplasmacytic infiltrate was identified, with a moderate CD138 positive plasma cell infiltration (Figure 6A) and abundant IgG4 positive cells (Figure 6B).

The postoperative course was uneventful. The patient started to take semi-fluid on the day after surgery. As there were no complications such as hemorrhage or bile leak, the drainage tube was removed on postoperative day 4 and the patient was discharged on postoperative day 5. The patient was followed up for 10 months. He did not take any drugs after surgery, his serum concentration of IgG4 was within the normal range, and other laboratory tests gave normal results.

## Discussion

Since Hamano *et al*. [8] first proposed a new term of systemic IgG4-associated autoimmune disease in 2001, ISD has become a novel clinicopathological disease entity. This is a systemic disease that can involve multiple organs, such as the pancreas, biliary duct, central nervous system, lacrimal or salivary glands, lungs, liver, kidneys, prostate gland, skin, arteries, lymph nodes. The key histopathologic findings are abundant IgG4 lymphoplasmacytic infiltrate, obliterative phlebitis, and an eosinophil infiltrate [1].

Autoimmune pancreatitis and IAC are the most common manifestations of ISD; IAC is the biliary manifestation of ISD, and is often associated with autoimmune pancreatitis. Although the diagnosis of IAC is often established based on a combination of clinical, serologic, radiological, and histologic findings, strict criteria for IAC are lacking [9]. Several diagnostic criteria have been proposed, following the initial Japanese consensus criteria for the diagnosis of autoimmune pancreatitis [10]. The HISORt (histology, imaging, serology, other organ involvement, and response to corticosteroid) criteria [11] for autoimmune pancreatitis and the variant criteria for IAC [12], as well as the Asian consensus criteria [13] have been the most commonly applied criteria for diagnosing autoimmune pancreatitis and IAC. Limited epidemiologic data exist on the rarity of IAC. Studies have reported that IAC is found mostly in men older than 50 years [9]. Notwithstanding the fact that the clinical presentation of patients with IAC is highly variable, obstructive jaundice is most common, present in up to 75% of patients [9]. Patients may present with other symptoms, such as abdominal pain, weight loss, pruritus, and biochemical signs of pancreatitis and cholestasis [12].

The low clinical specificity makes it difficult to distinguish IAC from cholangiocarcinoma. Although a serum IgG4 increase is characteristic of IAC, it may not be diagnostic of the disease. The sensitivity and accuracy of serum IgG4 for ISD were reported as 50% and 60%, respectively [14]. According to a recent study from the Mayo Clinic, which investigated the ability of IgG4 to distinguish IAC from cholangiocarcinoma reliably, serum IgG4 levels more than twice the upper limits of normal provided a specificity of 97% and a sensitivity of 50% in distinguishing IAC from cholangiocarcinoma [15]. Nevertheless, an IgG4 increase does occur in the absence of ISD, and hence increased serum IgG4 levels alone should not be used to diagnose IAC. Serum CA 19-9 levels can also be of use because concentrations greater than 100 IU/ml are less likely in IAC than in cholangiocarcinoma (18% versus 60% to 80%) [16, 17]. In addition, biliary strictures in IAC also do not have any highly specific diagnostic features, unlike autoimmune pancreatitis, in which typical pancreatic imaging features, such as diffuse sausage-shaped pancreatic enlargement and diffuse irregular narrowing of the pancreatic duct, have been described. Imaging often shows proximal bile duct or intrahepatic strictures with dilatation of the upstream biliary system, along with sclerosing changes, which are quite similar to cholangiocarcinoma. This indicates that, while imaging modalities are useful in determining the level of bile duct obstruction, they are limited when it comes to establishing a definitive diagnosis, distinguishing IAC from malignant biliary strictures reliably [18, 19].

An unexplained biliary stricture, not caused by trauma or choledocholithiasis, is often presumed to be caused by malignancy. In our case, with normal serum concentrations of IgG4 (1.86 g/l) and apparently elevated levels of CA19-9, the images showing segmental biliary stricture and no other organ involvement were assumed to indicate ISD; it is extremely difficult to distinguish IAC from cholangiocarcinoma preoperatively. Although endoscopic retrograde cholangiopancreatography is the most commonly performed procedure for cholangiocarcinoma and can provide a tissue diagnosis through brush cytology of the bile duct, the sensitivity of biliary brush cytology to diagnose cholangiocarcinoma may be as low as 30% [20]. Although endoscopic ultrasonography can complement the role of endoscopic retrograde cholangiopancreatography and provide a tissue diagnosis through fine needle aspiration and staging through ultrasound imaging, it can also lead to tumor seeding [20]. Hence, choledochectomy with Roux-en-Y hepaticojejunostomy was performed.

The laparoscopic technique is indispensable in biliary surgery. Compared with traditional open surgery, most studies have reported that laparoscopic techniques can achieve better cosmesis, shorter hospital stay, and faster postoperative recovery [21–24]. By choosing the laparoscopic approach, not only are the incision complications minimized, but also the quality of visualization is significantly enhanced, leading to greater precision in the control of reconstructions. However, it is important to have an experienced hand with wonderful skills. As with open surgery, laparoscopic ductoplasty appears to be technically possible in experienced hands. A proper wide hepaticojejunostomy must be performed for sufficient bile drainage. After surgery, the anastomotic stoma would become smaller because of inflammation and denuded epithelial mucosa of the bile duct. The posterior wall anastomosis is the most challenging step. Based on our experience, keeping the long corner stay sutures at the 3 o’clock and 9 o’clock positions of the anastomotic stoma was able to maintain tension to provide a clear view of posterior wall and allow more precise anastomosis. Maintaining the integrity of this anastomosis is important, and lessening the tension of the anastomosis is also a key point in preventing the occurrence of bile leakage. The intestinal mesentery should be isolated from the mesenteric root as much as possible, to ensure appropriate mobility of the biliary limb. In addition, the greater omentum should be separated if necessary. Moreover, interrupted sutures of seromuscular layer are also helpful, to reduce tension.

## Conclusions

Our case suggests that totally laparoscopic Roux-en-Y hepaticojejunostomy using an intracorporeal hand-sewn technique is a feasible procedure. Adequate preoperative evaluation, appropriate intraoperative hand-sewn techniques, and highly skilled laparoscopic techniques are the key factors of success in laparoscopic hepaticojejunostomy.

## Consent

Written informed consent was obtained from the patient for the publication of this case report and any accompanying images.

## Abbreviations

HISORt: histology, imaging, serology, other organ involvement, and response to corticosteroid
IAC: IgG4-associated cholangitis
IgG4: immunoglobulin G4
ISD: IgG4-associated systemic disease.
